# Tamoxifen-Independent Recombination in the *RIP-CreER* Mouse

**DOI:** 10.1371/journal.pone.0013533

**Published:** 2010-10-22

**Authors:** Yanmei Liu, Jakob Suckale, Jimmy Masjkur, Maria Grazia Magro, Anja Steffen, Konstantinos Anastassiadis, Michele Solimena

**Affiliations:** 1 Molecular Diabetology, Paul Langerhans Institute Dresden, School of Medicine and University Clinic ‘Carl Gustav Carus’, Dresden University of Technology, Dresden, Germany; 2 Max Planck Institute of Molecular Cell Biology and Genetics, Dresden, Germany; 3 Medical Clinic III, University Clinic ‘Carl Gustav Carus’, Dresden University of Technology, Dresden, Germany; 4 Genetic Engineering of Stem Cells, BioInnovations Zentrum, Dresden University of Technology, Dresden, Germany; 5 Center for Regenerative Therapies Dresden, Dresden University of Technology, Dresden, Germany; University of Tor Vergata, Italy

## Abstract

**Background:**

The inducible Cre-lox system is a valuable tool to study gene function in a spatial and time restricted fashion in mouse models. This strategy relies on the limited background activity of the modified Cre recombinase (CreER) in the absence of its inducer, the competitive estrogen receptor ligand, tamoxifen. The *RIP-CreER* mouse (*Tg (Ins2-cre/Esr1) 1Dam*) is among the few available β-cell specific *CreER* mouse lines and thus it has been often used to manipulate gene expression in the insulin-producing cells of the endocrine pancreas.

**Principal Findings:**

Here, we report the detection of tamoxifen-independent Cre activity as early as 2 months of age in *RIP-CreER* mice crossed with three distinct reporter strains.

**Significance:**

Evidence of Cre-mediated recombination of floxed alleles even in the absence of tamoxifen administration should warrant cautious use of this mouse for the study of pancreatic β-cells.

## Introduction

The inducible Cre-lox system has become an important tool for the conditional deletion or expression of genes of interest within a given cell type [1]. For this reason it has been widely used for lineage tracing of cells during embryonic development and in adult mice [2]. These studies typically require two mouse lines: a mouse in which the inducible Cre recombinase is driven by a tissue/cell-specific promoter and the target or reporter mouse, in which the desired gene contains two loxP sites for Cre-mediated recombination [1]. Specifically, the bacteriophage P1 Cre recombinase catalyzes site-specific recombination between two loxP sites and efficiently excises DNA flanked by two loxP sites in the same orientation [3]. Cre recombinase was made inducible by fusing it with the ligand-binding domain (LBD) of the estrogen receptor (ER) [4]. In the absence of its ligand, CreER is inactive as it is confined to the cytoplasm, via binding to heat shock protein-90. Upon estrogen binding, CreER moves into the nucleus where it engages its DNA targets. To avoid the unwanted effects of endogenous estrogen binding, the estrogen LBD has been further mutated, leading to the generation of several CreER recombinases that are sensitive to the synthetic ligand 4-hydroxytamoxifen (4-OHT) but not to estradiol [5]. For example, the CreER^T^ recombinase contains the human ER-LBD with a G521R mutation [5], while CreER^TM^ contains the mouse ER-LBD with a G525R mutation [6]. Different versions of CreER are being developed to decrease their nuclear translocation in the absence of the inducer, while increasing their sensitivity to tamoxifen and their recombination efficiency. More recent and useful CreER variants include MerCreMer, a double fusion of Cre with two ER^TM^ domains [7], and CreER^T2^ (G400V/M543A/L544A triple mutation of human ER-LBD) [8]. The complete absence of nuclear CreER without tamoxifen induction is crucial for conditional systems, because even the effects of a minor leakiness will accumulate over time due to the irreversibility of every recombination event. Cell adaptation to the altered gene expression pattern induced by the CreER recombinase, even in the absence of tamoxifen, can complicate further the analysis of gene function or cell lineage tracing studies. Therefore, the availability of mouse strains with tightly controlled CreER activity is highly desirable.

The *RIP-CreER* mouse (*Tg (Ins2-cre/Esr1) 1Dam*) was originally reported in 2004 and employed to investigate the origin of newly generated insulin-producing cells (β-cells) in adult mice and following partial pancreatectomy [9]. In this line, the use of the rat insulin promoter 2 to drive the expression of CreER restricts the expression of the recombinase to pancreatic β-cells. To further study the biology of pancreatic β-cells we crossed this *RIP-CreER* mouse with 3 Cre-target lines, including a Rosa26 knock-in mouse (*R26-Stop-HA3-ICA512-CCF*) for the conditional overexpression of the cleaved cytosolic fragment of ICA512 (ICA512-CCF) [10], a *Rosa26-lacZ* reporter mouse [11] and a *PTBP1^loxP/loxP^* line for the conditional knockout of PTBP1 [12]. Our data indicate that in all three cases recombination of the Cre-target genes was triggered very early on, in the absence of tamoxifen treatment.

## Results

### Tamoxifen-independent recombination in the Rosa26 locus of *RIP-CreER* mice

**Figure 1:**
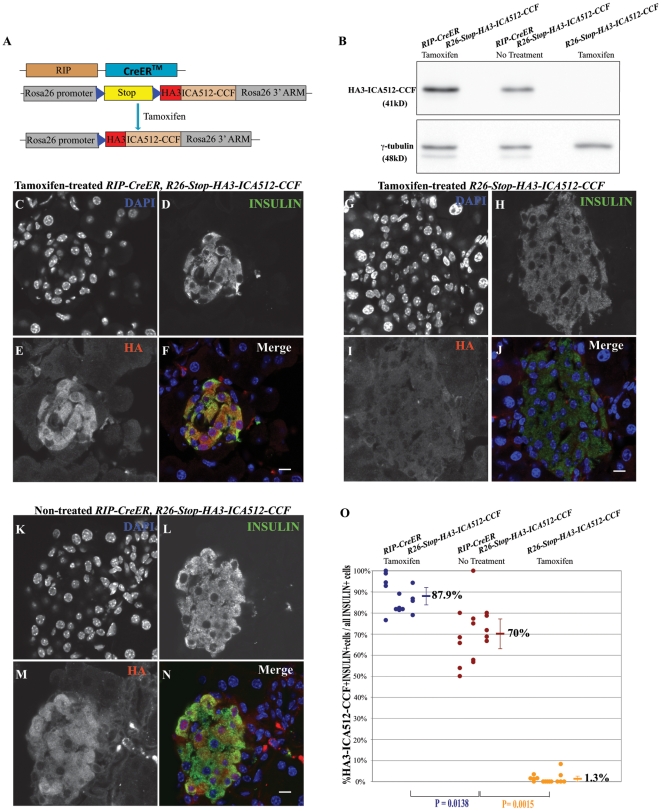
Tamoxifen-independent expression of *HA3-ICA512-CCF* in β-cells of *RIP-CreER, R26-Stop-HA3-ICA512-CCF* mice. **A**. Strategy for the generation of the inducible *R26-Stop-HA3-ICA512-CCF* knock-in mouse line. **B**. Western blotting of pancreatic islet extracts from 3-month-old tamoxifen-treated or untreated *RIP-CreER, R26-Stop-HA3-ICA512-CCF* mice or tamoxifen-treated *R26-Stop-HA3-ICA512-CCF* mice with anti-HA and anti-γ-tubulin antibodies (similar results were obtained from 3 independent experiments). **C-N**. Confocal microscopy images of pancreatic cryosections from 4-month-old tamoxifen-treated *RIP-CreER, R26-Stop-HA3-ICA512-CCF* (C-F) and *R26-Stop-HA3-ICA512-CCF* (G-J) and untreated *RIP-CreER, R26-Stop-HA3-ICA512-CCF* (K-N) mice stained with DAPI (C, G and K), as well as with anti-insulin (D, H and L) and anti-HA antibodies (E, I and M). Triple stainings (merge) are shown in F, J and N. Scale bars in F, J and N equal 10 µm. **O.** Quantification and statistical analysis of HA3-ICA512-CCF-positive cells relative to total insulin-positive cells in tamoxifen-treated or untreated *RIP-CreER, R26-Stop-HA3-ICA512-CCF* mice, and tamoxifen-treated *R26-Stop-HA3-ICA512-CCF* mice. Dots represent individual islets from 3 mice in each genotype group. The p value of a t-test between groups is shown.

To study the function of ICA512-CCF *in vivo*, we generated the *R26-Stop-HA3-ICA512-CCF* knock-in mouse, in which three-HA-tagged *ICA512-CCF* preceded by a floxed Stop cassette was introduced into the Rosa26 locus. The *R26-Stop-HA3-ICA512-CCF* mouse was then crossed with the *RIP-CreER* mouse [9] **(** **Fig. 1A** **)**. In principle, in the progeny containing both transgenes, the β-cell restricted expression of HA3-ICA512-CCF should have been detectable only upon tamoxifen-induced removal of the floxed Stop cassette **(** **Fig. 1A** **)**. Western blotting with an anti-HA antibody, however, revealed the expression of HA3-ICA512-CCF in islets isolated from 3-month-old *RIP-CreER*, *R26-Stop-HA3-ICA512-CCF* mice regardless of tamoxifen treatment **(** **Fig. 1B** **)**. In the control, HA3-ICA512-CCF was not detected in the extract of islets from *R26-Stop-HA3-ICA512-CCF* single transgenic littermates **(** **Fig. 1B** **)**. Cryosections of pancreatic tissue from 3-and 4-month-old mice were examined by immunofluorescence microscopy using anti-HA and anti-insulin antibodies. Immunoreactivity for HA was readily detectable in insulin-positive cells, i.e. in β-cells, of tamoxifen-treated *RIP-CreER*, *R26-Stop-HA3-ICA512-CCF* mice (**Fig. 1C-F**). The specificity of this labeling was confirmed by the absence of immunoreactivity for HA in pancreatic sections of tamoxifen-treated *R26-Stop-HA3-ICA512-CCF* mice (**Fig. 1G-J**). However, a positive reactivity for HA was also present in pancreatic sections of *RIP-CreER*, *R26-Stop-HA3-ICA512-CCF* mice that had not been treated with tamoxifen (**Fig. 1K-N**). Quantification of 15 islets from 3 mice in each genotype group revealed that at 4-month age, 70%±12.8% (mean ± SD) of the insulin positive cells were also positive for HA3-ICA512-CCF in non-treated *RIP-CreER*, *R26-Stop-HA3-ICA512-CCF* mice compared to 87.9%±7.4% in tamoxifen-treated *RIP-CreER*, *R26-Stop-HA3-ICA512-CCF* mice (**Fig. 1O**). Taken together, these data indicate that in these mice nuclear translocation of CreER occurred even in the absence of tamoxifen.

**Figure 2:**
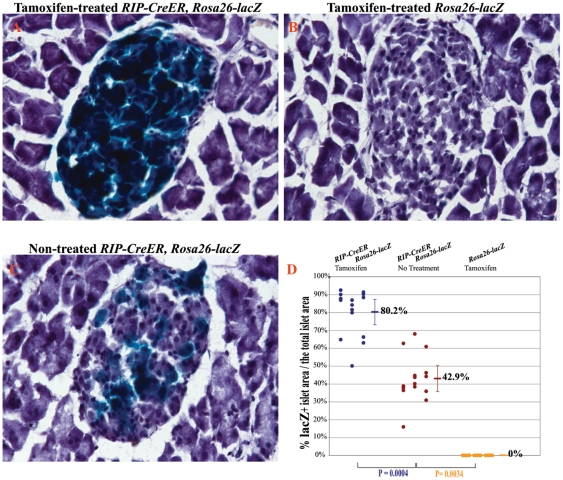
β*-galactosidase* gene (*lacZ*) expression in *RIP-CreER, Rosa26-lacZ* mice. **A-C.** LacZ staining of the pancreatic tissue from 10-week-old tamoxifen-treated *RIP-CreER, Rosa26-lacZ* mice (A), and *Rosa26-lacZ* (B) and untreated *RIP-CreER, Rosa26-lacZ* (C) mice. **D.** Quantification and statistical analysis of the lacZ-positive area relative to the total islet area in tamoxifen-treated or untreated *RIP-CreER, Rosa26-lacZ* mice, and tamoxifen-treated *Rosa26-lacZ* mice. Dots represent individual islets from 3 mice in each genotype group. The p value of a t-test between groups is shown.

To evaluate whether the tamoxifen-independent expression of HA3-ICA512-CCF was a specific limit of the *RIP-CreER, R26-Stop-HA3-ICA512-CCF* mouse, we then bred *RIP-CreER* mice to *Rosa26-lacZ* mice, in which the β*-galactosidase* gene (*lacZ*) preceded by a floxed Stop cassette is inserted in the Rosa26 locus [11]. In 10-week-old, tamoxifen-untreated *RIP-CreER*, *Rosa26-lacZ* mice 42.9%±13.1% of the islet area was lacZ positive (**Fig. 2C, 2D**) compared to 80.2%±12.9% upon tamoxifen-treatment (**Fig. 2A, 2D**). Conversely, no lacZ-positive islets were found in tamoxifen-treated *Rosa26-lacZ* littermates (**Fig. 2B, 2D**). These findings corroborate the conclusion that the *RIP-CreER* mouse displays a tamoxifen-independent recombinase activity, at least toward transgenes introduced in the Rosa26 locus.

### Tamoxifen-independent recombination outside of the Rosa26 locus in the *RIP-CreER* mouse

**Figure 3:**
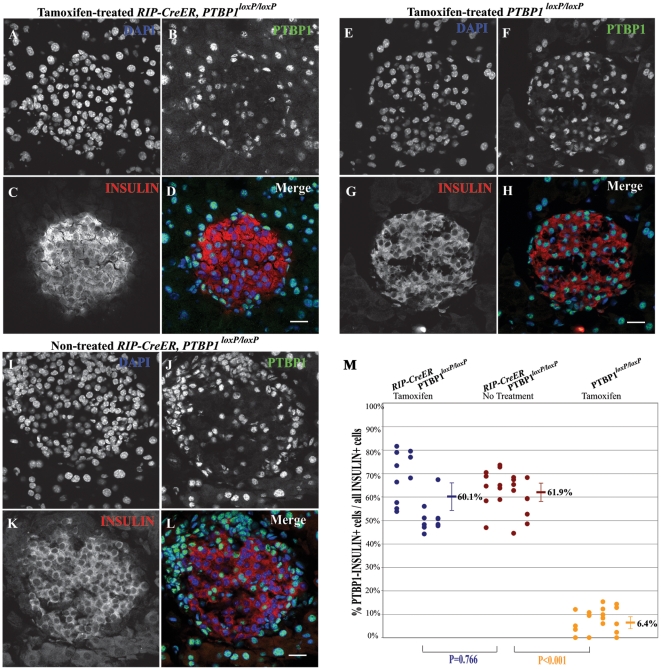
Tamoxifen-independent knockout of *PTBP1* in adult β-cells of *RIP-CreER, PTBP1^loxP/loxP^* mice. **A-L**. Confocal microscopy images of pancreatic cryosections from 8-week-old tamoxifen-treated *RIP-CreER, PTBP1^loxP/loxP^* (A-D) and *PTBP1^loxP/loxP^* (E-H) and untreated *RIP-CreER, PTBP1^loxP/loxP^* (I-L) mice stained with DAPI (A, E and I), as well as with anti-PTBP1 (B, F and J) and anti-insulin antibodies (C, G and K). Triple stainings (merge) are shown in D, H and L. Scale bars in D, H and L equal 20 µm. **M.** Quantification and statistical analysis of PTBP1-positive cells relative to total insulin-positive cells in tamoxifen-treated and untreated *RIP-CreER*, *PTBP1^loxP/loxP^* mice, and tamoxifen-treated *PTBP1^loxP/loxP^* mice. Dots represent individual islets from 4 mice in each genotype group. The p value of a t-test between groups is shown.

To evaluate whether the constitutive Cre activity detected in *RIP-CreER* mice is limited to the Rosa26 locus, we analyzed *RIP-CreER, PTBP1^loxP/loxP^* mice. PTBP1 (polypyrimidine tract-binding protein 1) [12] is an RNA-binding protein that, in insulinoma INS-1 cells, enhances the stability and translation of mRNAs encoding insulin granule proteins [13]. The insertion of two loxP sites flanking exons 3 to 7 of *PTBP1* allows the deletion of this gene (Suckale et al., in preparation). Pancreatic sections of 8-week-old *RIP-CreER*, *PTBP1^loxP/loxP^* mice treated or untreated with tamoxifen were examined by immunocytochemistry for the expression of insulin and PTBP1. In tamoxifen-treated *RIP-CreER, PTBP1^loxP/loxP^* mice 60∼1%±12.6% of the insulin positive cells were negative for PTBP1 compared to 61.9%±8.4% in the case of tamoxifen untreated mice (**Fig. 3A-D, 3I-M**). In contrast, virtually all β-cells of tamoxifen treated *PTBP1^loxP/loxP^* mice were PTBP1 positive (**Fig. 3E-H, 3M**). Thus, Cre-induced knockout of *PTBP1* occurred in most β-cells regardless of tamoxifen-treatment.

### Subtle Tamoxifen-independent recombination in the Rosa26 locus of *Pdx1-CreER* mice

**Figure 4:**
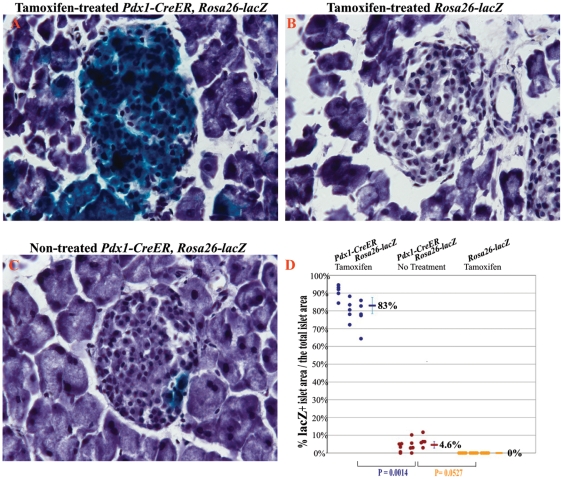
β*-galactosidase* gene (*lacZ*) expression in *Pdx1-CreER, Rosa26-lacZ* mice. **A-C.** LacZ staining of the pancreatic tissue from 4-month-old tamoxifen-treated *Pdx1-CreER, Rosa26-lacZ* mice (A), and *Rosa26-lacZ* (B) and untreated *Pdx1-CreER, Rosa26-lacZ* (C) mice. **D.** Quantification and statistical analysis of the lacZ-positive area relative to the total islet area in tamoxifen-treated or untreated *Pdx1-CreER, Rosa26-lacZ* mice, and tamoxifen-treated *Rosa26-lacZ* mice. Dots represent individual islets from 3 mice in each genotype group. The p value of a t-test between groups is shown.

Next, we evaluated the tamoxifen-independent recombination of *Pdx1-CreER* mouse [14]. Upon crossing this mouse with the *Rosa26-lacZ* mouse employed previously, we detected tamoxifen-independent X-gal staining only in few β-cells of 2-, 4- and 6-month-old mice (**Fig. 4A-D** **and Data not shown**). Morphometric analysis, in particular, showed that in 4-month-old tamoxifen-untreated *Pdx1-CreER*, *Rosa26-lacZ* mice only 4.6%±3.4% of the islet area was lacZ positive compared to 83%±8.3% upon tamoxifen treatement (**Fig. 4D**).

## Discussion

In the original study the *RIP-CreER* was bred with a Z/AP reporter strain [9]. The authors wrote that: “by 10 months of age, double transgenic mice that did not receive tamoxifen had occasionally one to five HPAP^+^ cells per islet, reflecting the cumulative lifetime leakage of the system.” [9]. In our case the leakiness of the system was clearly stronger. Its tamoxifen-independent recombinase activity was detected in 3 different reporter/target mice, 2 loci and was already pronounced at 2 months of age. There are at least two possible explanations for these discrepancies. One possibility is that after many generations of backcrossing to the C57BL/6 background, the expression of the randomly integrated RIP-CreER transgene is enhanced, thus increasing the probability of its nuclear entry, even in tamoxifen-untreated cells. Another more likely possibility is that the Z/AP cassette in the Z/AP reporter mouse strain is less prone to recombination, i.e. it is inserted in an area of the chromatin less amenable to recombination. Easy access of the CreER to the target locus may result in both high recombination efficiency and leakiness of the system as seen in the 3 lines described here. On the other hand, recombination of a less accessible, heterochromatic locus could be less efficient, but also less leaky, as in the case of the Z/AP mouse.

One way to mitigate such problems is the use of the *Pdx1-CreER* mouse, which was also generated in the Melton lab [14]. Upon its crossing with the *Rosa26-lacZ* mouse, we found that in this mouse tamoxifen-independent recombination is a relative rare event. As both *Pdx1-CreER* and *RIP-CreER* mice were generated using the CreER^TM^ variant, the tighter phenotype of the former is conceivably due to the lower activity of the *Pdx-1* promoter relative to the *RIP* promoter, resulting in lower expression of CreER^TM^. Buelow and Scharenberg have recently shown in cell lines that the ligand-independent activity of inducible Cre correlates with its level of expression [15]. Hence, CreER transgenic mice should be carefully evaluated regarding the activity of the promoter and the protein levels of CreER. The study of β-cells could also benefit from the development of additional driver mice in which a single copy of Cre fused to one or even two ER^T2^ domains is inserted downstream of a β-cell specific promoter, such as insulin, using BAC-based recombination technologies.

In conclusion, we observed tamoxifen-independent recombination in the *RIP-CreER* mouse already in early adult age. Investigators working in the field of islet biology should be aware of this potential hurdle when planning the employment of this mouse for their studies.

## Materials and Methods

### Ethics statement

All animal experiments were conducted in a licensed animal facility in accordance with the *German Animal Welfare Act*, following the guidelines of the *European Convention for the Protection of Vertebrate Animals Used for Experimental and Other Scientific Purposes* and approved by the regional council.

### Generation of the *R26-Stop-HA3-ICA512-CCF* mouse

Primers NheI-SA-F-46 (5′- attatagctagcgtgacctgcacgtctagggcgcagtagtccaggg -3′) and NheI-SA-MCS-R-117 (5′-Tataatgctagcacgcgtatataattcccgggttcgaatctagaatataattccgcggac tggaaagaccgcgaagagtttgtcctcaaccgcgagctgtggaaaaaaaagggacag -3′) were used to amplify SA-MCS (Splice Acceptor and Multiple Cloning Site) fragment using the plasmid pMB80 (R26-CreER, from Addgene) as template. SA-MCS (digested with NheI) was cloned into the pROSA26-1 vector (plasmid pMB80 digested with XbaI) to obtain the pROSA26-SA-MCS construct. XhoI and ClaI were used to remove PGK-DTA from pROSA26-SA-MCS and obtain pROSA26-SA-MCS-dPGK-DTA. Primers SacII-r-loxP-F (attataccgcggataacttcgtatagcatacattatacgaagttattaggtccctcgacctgcaggaattc) and NheI-r-loxP-R (ttattagctagctataacttcgtataatgtatgctatacgaagttatattaagggttccggatcagcttgatgg) were used to amplify the loxP-Stop-loxP fragment from pPGKneotpAlox2 (from Addgene). The loxP-Stop-loxP (digested with SacII and NheI) fragment was then ligated with pROSA26-SA-MCS-d-PGK-DTA (digested with SacII and XbaI) to obtain pROSA26-Stop. HA3-ICA512-CCF [16] (digested with EcoRI and MluI) was ligated with HSV-TK-3′UTR (digested with MfeI and MluI), which was amplified with primers MfeI-HSV-TK-3′UTR-F and MluI-HSV-TK-3′UTR-R from pEGFPN1. HA3-ICA512-CCF-HSV-TK-3′UTR (digested with AgeI and MluI) was then cloned into pROSA26-Stop (digested with XmaI and MluI) to obtain the final construct pR26-Stop-HA3-ICA512-CCF (GenBank accession number HM588138). All the constructs were confirmed to be correct by sequencing. pR26-Stop-HA3-ICA512-CCF was digested by PacI and KpnI and then electroporated into R1 embryonic stem (ES) cells (129 genetic background), which were then selected for resistance to G418. The targeted ES cells in which the floxed Stop cassette and HA3-ICA512-CCF sequences were correctly inserted and exclusively present in the Rosa26 locus were identified by Southern blots with 5′-arm, 3′-arm and internal probes. The ES cells were injected into eight-cell mouse embryos to generate the *R26-Stop-HA3-ICA512-CCF* mouse founder. Primers pRosa26-1-757-77 (5′-gtttccgacttgagttgcctc-3′) and unrec-R (5′- ccacttgtgtagcgccaagtg-3′) were used for PCR-genotyping of mice. The mouse *R26-Stop-HA3-ICA512-CCF* line was kept in the 129X1 SvJ genetic background.

### Generation of conditional PTBP1 knockout mouse

In the *PTBP1^loxP/lopP^* mouse, two loxP sites flank exons 3 to 7 of *PTBP1*. The detailed information regarding this mouse generation is described elsewhere (Suckale et al., in preparation). The GenBank accession number of the related construct is HM588137.

### Tamoxifen treatment

3–5 mg tamoxifen (Sigma, Cat# T-5648) were fed to the mice by gavage, every other day and 3 times in total.

### Immunohistochemistry

Cryosections were re-hydrated in PBS, permeabilized with 0.3% Triton X-100 in PBS for 15 min, and incubated for 1 hour in blocking buffer (goat serum dilution buffer). Sections were incubated with primary antibodies in the blocking buffer overnight at 4°C, washed with PBS, then further incubated with the appropriate fluorochrome-conjugated secondary antibodies raised in goat for 1 hour. Sections were finally rinsed with water and mounted using Moviol solution. Primary antibodies included a mouse anti-HA antibody (Roche), a guinea pig anti-insulin antibody (Gene Tex), and a mouse anti-PTBP1 antibody (Zymed). Confocal images were acquired on a Zeiss LSM 510 microscope. Total number of insulin positive cells as well as insulin/HA3-ICA512-CCF double positive cells or insulin positive/PTBP1 negative cells were counted manually from images processed with the Fiji software [17]. The percentage and the p value of a t-test between groups were calculated with Microsoft Excel.

### Staining for β-galactosidase activity

Mouse pancreatic cryosections were fixed with 0.2% glutaraldehyde buffer (for 50 ml, 0.5 ml 0.5 M EGTA, pH 7.3; 5.0 ml 1 M MgCl2; 44.1 ml PBS; 0.4 ml 25% glutaraldehyde), and then washed 3 times for 15 min in washing buffer (for 500 ml, 1.0 ml 1 M MgCl2; 5.0 ml 1% Sodium deoxycholate (NaDOC); 5.0 ml 2% Nonidet-P40; 489 ml PBS). The sections were further incubated with staining buffer (for 100 ml, 4.0 ml 25 mg/ml X-gal (5-bromo-4-chloro-3-indoxy-β-D-galactopyranoside)); 0.21 g K-ferrOcyanide; 0.16 g K-ferrIcyanide in washing buffer) in a dark chamber at 30°C overnight. The sections were counterstained with haematoxylin, dehydrated through ethanol series and mounted with Depex mounting medium.

### Quantification of lacZ positive islet area in the islet

Images of sections stained for lacZ were taken with a Zeiss Observer Z1 using a 40X objective. The images were then analyzed with Adobe Photoshop and Fiji to measure the lacZ positive islet area relative to the total islet area.

### Islet isolation and western blot

Pancreatic islets were isolated as previous described [18]. After 24-hour culture, islets were harvested at 4°C in cell lysis buffer to obtain total extracts. 10 mg protein was separated by 10% SDS-PAGE and immunoblotted with a rabbit anti-HA antibody (Abcam) and a mouse anti-γ-tubulin antibody. The blot was developed using the Supersignal West Pico or Femto kits (Pierce) and Chemiluminescence was detected with a LAS 3000 Bioimaging System (Fuji, Tokyo, Japan).
